# ENPP2 Dysregulation Defines a Candidate Biomarker Axis Coupling Tumor‐Intrinsic cAMP Signaling to Macrophage Polarization in Hepatocellular Carcinoma

**DOI:** 10.1155/humu/9266306

**Published:** 2026-05-23

**Authors:** Shiyi Chen, Jinli Zheng, Jianwu Long, Peng Ou, Jian Tong, Fan Lin

**Affiliations:** ^1^ Department of Hepatobiliary Surgery, The First Affiliated Hospital of Jinan University, Guangzhou, Guangdong, China, jd120.com; ^2^ Department of Hepatobiliary Surgery, The Affiliated Nanhua Hospital, Hengyang Medical School, University of South China, Hengyang, Hunan, China, usc.edu.cn

**Keywords:** cAMP signaling, ENPP2, hepatocellular carcinoma, macrophage polarization, tumor-associated macrophages

## Abstract

Hepatocellular carcinoma (HCC) shows substantial biological heterogeneity and commonly develops within an immunosuppressive microenvironment. Tumor‐associated macrophages (TAMs), particularly M2‐skewed subsets, are repeatedly associated with aggressive disease and may represent a biologically meaningful phenotype for biomarker development. Ectonucleotide pyrophosphatase/phosphodiesterase 2 (ENPP2) has been implicated in malignant behavior, but its linkage to TAM polarization and its potential as a laboratory‐interpretable translational readout in HCC remain insufficiently clarified. ENPP2 expression was examined in paired HCC and adjacent tissues and referenced to public cohorts to provide clinical context. ENPP2 was overexpressed or silenced in HCC cells, followed by assays of proliferation, apoptosis, migration, and invasion. Macrophage polarization was evaluated using a noncontact Transwell coculture system with marker assessment and flow‐cytometric readouts. Intracellular cyclic adenosine monophosphate (cAMP) was quantified, and forskolin was used to interrogate pathway involvement. Xenograft experiments were conducted to examine in vivo tumor growth. ENPP2 was upregulated in HCC and showed an association with unfavorable outcomes in public datasets. ENPP2 increased malignant phenotypes in HCC cells and shifted cocultured macrophages toward an M2‐skewed state, whereas ENPP2 suppression produced the opposite pattern. ENPP2 modulation coincided with changes in intracellular cAMP signaling, and forskolin partially attenuated phenotypes observed after ENPP2 knockdown. These findings delineate a novel ENPP2/cAMP signaling axis that directly links tumor‐intrinsic ENPP2 overexpression in HCC cells to the induction of M2‐skewed TAM polarization, a mechanism that has not been previously characterized in HCC. This work not only identifies ENPP2 as a dual regulator of HCC cell malignancy and TAM polarization but also establishes cAMP as the critical intracellular mediator of this crosstalk, thereby strengthening the logical connection between these elements and advancing the understanding of HCC tumor‐immune microenvironment interactions beyond existing literature.

## 1. Introduction

As a major global health challenge, hepatocellular carcinoma (HCC) imposes a significant burden worldwide. It represents the third leading cause of cancer death [1]. Epidemiological data from 2020 highlight a pronounced burden in China, contributing 55% (approximately 410,000) of the 800,000 global new cases and suffering 391,000 fatalities, which places HCC second among all cancer‐related mortalities in China [2, 3]. Despite therapeutic advancements, including targeted combination immunotherapies and monoclonal antibodies that have improved relapse‐free survival and patient outcomes, the overall prognosis remains unfavorable [4–6]. Persistent limitations of current treatments underscore the need to identify actionable molecular targets.

Functioning as a secreted enzyme, ectonucleotide pyrophosphatase/phosphodiesterase 2 (ENPP2/Autotaxin) primarily facilitates the hydrolysis of lysophospholipids into lysophosphatidic acid (LPA) [7, 8]. This glycoprotein, a member of the ecto‐phosphodiesterase family [9] and encoded on Chromosome 8, generates LPA, a lipid mediator that plays crucial roles in proliferation, migration, and angiogenesis. Prior research indicates that the ENPP2 inhibitor GLPG1690 enhanced survival in a murine colorectal cancer model by modifying intestinal barrier integrity and gut microbiota [10]. Li and colleagues reported that ENPP2 silencing reduced tumor burden and bone resorption markers in a multiple myeloma xenograft model, supporting its protumorigenic role [11]. Research further indicates that exosomal miR‐29c‐3p released by M1 macrophages can suppress the malignant potential of melanoma cells through its suppression of ENPP2 [12]. These findings together indicate a widespread involvement of ENPP2 in oncogenesis, presenting it as a promising target for therapy, even though its detailed actions and pathways in HCC are not fully mapped.

Within the tumor microenvironment (TME), a considerable proportion of immune cells are tumor‐associated macrophages (TAMs). Influenced by various components of the TME such as signaling molecules, these macrophages can adopt different functional phenotypes, commonly categorized as the antitumorigenic M1 or the tumor‐promoting M2 state [13, 14]. Notably, polarization is dynamic, skewing toward the M2 subtype as malignancy advances, thereby fostering an immunosuppressive milieu conducive to tumor growth and dissemination [15]. For instance, loss of the small ubiquitin‐like modifier (SUMO)‐specific protease sentrin‐specific protease 3 (SENP3) was found to promote M2 polarization of bone marrow‐derived macrophages (BMDM), accelerating breast cancer progression [16]. Clinical analyses in colorectal cancer have associated high M2 macrophage density with poorer cancer‐specific survival, underscoring the prognostic relevance of macrophage polarization [17]. In HCC, hypoxia‐induced protein associated with AP‐1 regulation of histone acetylation (PAARH) overexpression was reported to enhance vascular endothelial growth factor (VEGF) expression, driving M2 polarization and facilitating immune evasion [18]. Thus, TAM polarization critically influences HCC pathogenesis. Whether ENPP2 affects HCC cells in a manner that subsequently alters TAM polarization remains unclear.

Cyclic adenosine monophosphate (cAMP) serves as a pivotal intracellular secondary messenger, transducing extracellular signals to regulate diverse cellular responses [19]. In breast cancer, elevated cAMP levels have been linked to leptin‐induced apoptosis, suggesting crosstalk between these pathways [20]. The cAMP signaling cascade participates in various tumor‐promoting mechanisms across different cancers. To illustrate, the protein 7‐dehydrocholesterol reductase (DHCR7) facilitates the spread of bladder cancer by operating through the cAMP/protein kinase a (PKA)/focal adhesion kinase (FAK) signaling axis [21]. Similarly, elevated levels of GRIK5 boost the growth and invasive capacity of colon cancer cells via stimulation of the cAMP/PKA pathway and subsequent increase in cell adhesion molecule 3 (CADM3) expression [22]. Moreover, within non–small cell lung cancer, the activation of the takeda g protein‐coupled receptor 5 (TGR5 receptor) induces M2 polarization in macrophages, a process dependent on a signaling cascade involving cAMP, signal transducer and activator of transcription 3 (STAT3), and STAT6 [23].

While prior studies have established that tumor‐derived factors modulate TAM polarization in HCC, the specific role of ENPP2 and its downstream signaling cascade in linking HCC cell malignancy to TAM reprogramming remain unexplored. Additionally, cAMP signaling has been implicated in tumor progression and immune cell function, but its mechanistic link to ENPP2‐mediated TAM polarization in HCC is unknown. Integrating these observations, we hypothesized that ENPP2 might regulate the proliferative and metastatic behaviors of HCC cells and, via cAMP signaling, specifically and mechanistically influence TAM polarization. The objective of this work was to experimentally test this hypothesis, delineate the ENPP2/cAMP/TAM regulatory axis in HCC, and strengthen the logical connection between these elements, thereby advancing the understanding of HCC tumor‐immune crosstalk and identifying ENPP2‐centered molecular readouts for downstream translational evaluation.

## 2. Materials and Methods

### 2.1. Specimen Collection

In this study, clinical samples were collected and used in adherence to the ethical guidelines established in the Declaration of Helsinki. Prior ethical authorization was obtained from the Medical Ethics Committee of the University of South China (Approval No. 2024‐KY‐170). All participants provided written informed consent.

From 10 treatment‐naïve patients diagnosed with HCC in 2024, we collected tumor specimens along with matched nontumor tissues taken from a distance of 3–5 cm from the tumor edge. Each sample was split: One portion was snap‐frozen at −80°C, and the other was fixed in 4% paraformaldehyde (G1101, Servicebio, China).

### 2.2. GEPIA Database Analysis

The relevant data were obtained by specifying “ENPP2” as the gene of interest and “Hepatocellular carcinoma” as the cancer type for expression analysis using the GEPIA database (URL: http://gepia2.cancer‐pku.cn/#index, Version: GEPIA 2.0).

### 2.3. Culture Conditions and Pharmacological Interventions

The human HCC cell line HepG2 (CL‐0103, Procell, China) was maintained in minimum essential medium (MEM, M7278, Sigma‐Aldrich, United States) at 37°C within a humidified atmosphere containing 5% CO_2_. The MEM medium was enriched with 10% fetal bovine serum (FBS, A5669701, Gibco, United States), 1% penicillin‐streptomycin (15140122, Gibco, United States), and nonessential amino acids (NEAA, 11140050, Gibco, United States). To activate the cAMP pathway, a 24‐h treatment with 5‐*μ*M Forskolin (HY‐15371, MCE, United States) was administered to the cells.

THP‐1 monocytic cells (iCell‐h213, icell, China) were grown in the supplier‐recommended medium (iCell‐h213‐001b, icell, China) under identical culture conditions. To promote differentiation into macrophages, THP‐1 monocytic cells were exposed to 100 ng/mL phorbol 12‐myristate 13‐acetate (PMA, HY‐18739, MCE, United States) over a 48‐h period.

### 2.4. Cell Transfection

To manipulate ENPP2 expression, an overexpression plasmid and a specific shRNA were constructed. All oligonucleotide sequences were supplied by Anhui General Biologicals. For ENPP2 overexpression, a plasmid was engineered through the cloning of the complete ENPP2 coding sequence (Accession NM_006209) into the pcDNA3.0 vector. The short hairpin RNA (shRNA) sequences used are as follows: shENPP2: SS sequence: 5 ^′^‐GACTAGATATGATATCTTA‐3 ^′^, AS sequence: 5 ^′^‐TAAGATATCATATCTAGTC‐3 ^′^. shNC: 5 ^′^‐GTTCTCCGAACGTGTCACGT‐3 ^′^. Transfections were carried out employing Lipo6000 transfection reagent (C0526, Beyotime, China), with downstream assays commencing 48 h post‐transfection.

### 2.5. Cell Viability Assay (CCK‐8)

Approximately 5000 treated cells were added to each well of a 96‐well plate. Subsequent to cell adhesion, the medium was aspirated, and the wells were rinsed two to three times with phosphate‐buffered saline (PBS, 10010023, Gibco, United States). Next, CCK‐8 reagent (C0037, Beyotime, China) in fresh medium was applied to every well. Following a 30‐min period of incubation at 37°C under light‐protected conditions, the absorbance was quantified with a BioTek Synergy microplate reader.

### 2.6. Cell Formation Assay

To assess clonogenicity, a single‐cell suspension of transfected cells was prepared in complete medium at 3000 cells/mL and plated (1 mL/well) in six‐well plates. A 7‐day routine culture was maintained before examining the developed colonies (≥ 50 cells). The incubation was terminated by aspirating the medium, after which the cells were fixed in 4% paraformaldehyde. For the purpose of colony visualization and enumeration, crystal violet staining (C0121, Beyotime, China) was subsequently performed.

### 2.7. 5‐Ethynyl‐2 ^′^‐Deoxyuridine (EdU) Assay

Cell proliferative activity was evaluated with an EdU assay according to the manufacturer′s protocol (C0071S, Beyotime, China). After treatment, the medium was removed, and the cells were rinsed two to three times with PBS. A freshly prepared EdU working solution was then applied, and the cells were incubated for 2 h at 37°C before being fixed and permeabilized. To visualize EdU incorporation, the click reaction mixture was added and allowed to react for 30 min at room temperature in the dark. Following another two to three washes with PBS, cell nuclei were counterstained using DAPI (C1006, Beyotime, China). Image capture and analysis were performed on a fluorescence microscope (NIB600, Novel, China), which is equipped with a 10× eyepiece, 4×, 10×, 40×, and 100× objective lenses (numerical aperture: 0.25–1.25), a 12‐megapixel digital camera, and three fluorescence channels (DAPI, FITC, and TRITC) for multicolor fluorescence imaging.

### 2.8. Terminal Deoxynucleotidyl Transferase dUTP Nick End Labeling (TUNEL) Staining of Cells

TUNEL staining of cells was performed in accordance with the manufacturer′s protocol (C1086, Beyotime, China). After treatment, cells were rinsed with PBS (pH 7.4) to remove residual medium and metabolites. The cells were then fixed with 4% paraformaldehyde (G1101, Servicebio, China) at room temperature for 30 min, followed by permeabilization with freshly prepared permeabilization solution (0.3% Triton X‐100) for 5 min. The cells were incubated with a 50‐*μ*L working solution at 37°C for 60 min. After incubation, cells were washed three times with PBS (pH 7.4). DAPI (C1006, Beyotime, China) was diluted to 5 *μ*g/mL with PBS, added to each well, and incubated for 5 min in the dark to counterstain cell nuclei. Finally, fluorescence microscopy (NIB600, Novel, China) was employed to acquire images.

### 2.9. Cell Scratch

A confluent monolayer was created by seeding treated cells into six‐well plates. The confluent monolayer was wounded by drawing a linear scratch with a sterile pipette tip (200‐*μ*L volume). After washing with PBS to clear debris, serum‐free medium was replenished. Following a 24‐h incubation at 37°C, wound closure was evaluated and recorded by imaging.

### 2.10. Assessment of Cell Migration and Invasion Using Transwell System

Cell migration and invasion were analyzed with Transwell chambers (14141, Labselect, China) equipped with membranes of 8‐*μ*m pore size.

Migration assay protocol: Post‐transfection cells were harvested by trypsinization, rinsed, and reconstituted in medium without serum. The resulting suspension was transferred to the upper chamber of the Transwell apparatus. Meanwhile, 800 *μ*L of full growth medium containing 10% FBS was dispensed into the lower well to act as a chemical attractant. After incubating for 24 h, cells that did not traverse the membrane were gently removed from the top side. The cells which had migrated to the underside were immobilized with 4% paraformaldehyde, subjected to crystal violet staining, and their numbers were determined via microscopic examination.

Invasion assay protocol: In invasion experiments, the Transwell membrane was initially covered with a Matrigel layer (C0371, Beyotime, China) to mimic a physiological barrier. Cells, prepared in the same serum‐free manner, were plated onto the coated insert. The bottom chamber was supplied with 800 *μ*L of complete medium with serum to create a concentration gradient. Subsequent to a 24‐h incubation, cells failing to invade were cleared from the upper membrane surface with a cotton applicator. The invasive cells that penetrated the Matrigel were fixed in 4% paraformaldehyde (30 min, room temperature), stained with crystal violet, and then enumerated under a light microscope.

### 2.11. Coculture System

To assess the influence of HCC cells on macrophage polarization, we established a noncontact coculture model using Transwell inserts (14111, Labselec, Chinat) with a 0.4‐*μ*m pore size. We seeded macrophages (differentiated from THP‐1 cells via PMA treatment) into the lower well of a six‐well culture plate at a specific density of 5 × 10^4^ cells per well. Meanwhile, preconditioned HCC cells (after transfection and 48‐h culture to ensure stable ENPP2 expression) were plated in the upper chamber of a Transwell insert at a density of 2 × 10^5^ cells per well, resulting in a strict ratio of HCC cells to macrophages of 4:1. The insert was then transferred into the macrophage‐containing well, assembling the coculture setup. After 48 h of coculture, the polarization status of the macrophages residing in the lower compartment was analyzed.

### 2.12. Flow Cytometry for Macrophage Polarization

Following the coculture period, macrophages were harvested by trypsinization. After washing, the cell pellet was resuspended in flow cytometry staining buffer (BL1136A, Biosharp, China) and fixed at room temperature for 30 min. For immunophenotyping, an isotype control group was set up to eliminate nonspecific binding of antibodies and ensure the accuracy of flow cytometry data. The isotype control antibodies used were APC/Cy7‐conjugated mouse IgG isotype control (BioLegend, United States) and PE/Cy7‐conjugated mouse IgG isotype control (YEASEN, China), which were incubated with macrophages under the same conditions as the specific antibodies. Subsequently, cells in the experimental group were labeled in the dark at 37°C for 30–60 min using fluorescent antibodies: CD86‐APC/Cy7 (105030, BioLegend, United States) identified M1 macrophages, and CD206‐PE/Cy7 (32222ES25, YEASEN, China) identified M2 macrophages. Unbound antibodies were removed by two washing steps prior to resuspension and analysis on a BD Canto II flow cytometer. The flow cytometry data were gated based on the isotype control group to exclude nonspecifically stained cells, ensuring the reliability of the results.

### 2.13. Enzyme‐Linked Immunosorbent Assay (ELISA) for Detection of Macrophage Polarization‐Related Cytokines

ELISA assays were performed to detect the secretion levels of M1‐related cytokine interferon‐gamma (IFN‐*γ*) and M2‐related cytokine interleukin‐10 (IL‐10) in the coculture supernatant using commercial ELISA kits (Sangon, China) in accordance with the manufacturer′s instructions. The absorbance at 450 nm was measured using a BioTek Synergy microplate reader, and the cytokine concentrations were calculated according to the standard curve.

### 2.14. cAMP Measurement

Quantification of intracellular cAMP was performed based on the instructions provided with a commercially available ELISA kit (D770001, Sangon, China).

### 2.15. GST Pull‐Down Assay

The recombinant GST‐ENPP2 protein and a Flag‐tagged cAMP‐binding probe were obtained from Novoprotein. We conducted a GST pull‐down assay using the corresponding kit (abs50082, absin, China) and its protocol. Briefly, Glutathione MagBeads were equilibrated to room temperature and washed with resuspension buffer. For each reaction, 30 *μ*L of beads was aliquoted into two tubes designated as the control and experimental groups. The control tube received GST recombinant protein, whereas the experimental tube was incubated with GST‐ENPP2 protein. After incubation on a rotator at room temperature for 2 h, the beads were isolated magnetically, washed, and then mixed with 200 *μ*g of the Flag‐tagged probe for overnight incubation at 4°C with continuous rotation. On the following day, the beads were magnetically captured again and washed to eliminate nonspecific binding, and the specifically bound complexes were recovered by elution. The eluates were combined with loading buffer for subsequent analysis by western blot, with a parallel input sample prepared for comparison.

### 2.16. Xenograft Tumor Model

We obtained 4‐week‐old male and female BALB/c nude mice (approx. 18–20 g) from Jiangsu Huachuang Sino Biological Company. The mice were kept in an SPF facility with set conditions: 26°C, 50% ± 5% humidity, 12 h light/dark cycles, and ad libitum feeding. After a week of acclimatization, they were assigned to one of four groups, with three mice per group.

• pc‐NC: injected with vector‐transfected HCC cells
• pc‐ENPP2: injected with ENPP2‐overexpressing HCC cells
• sh‐NC: injected with scramble shRNA‐transfected HCC cells
• sh‐ENPP2: injected with ENPP2‐knockdown HCC cells

Cells were combined 1:1 with Matrigel, and a 200‐*μ*L suspension containing 6 × 10^6^ cells was administered via subcutaneous injection into the right flank. Tumor growth was tracked by caliper measurements every 2 days. For downstream evaluation, tumors were harvested on Day 21 following the sacrifice of the mice.

The experimental protocols were authorized by the Animal Ethics Committee at the University of South China (Approval No. 2025‐KY‐304). All procedures strictly followed the relevant institutional and national guidelines for the care and use of animals. Mice exhibiting severe adverse conditions (e.g., inability to feed or pronounced weight loss) were euthanized promptly via CO_2_ inhalation.

### 2.17. Hematoxylin and Eosin (H&E) Staining

Tumor tissues were fixed in 4% paraformaldehyde (24 h) and processed for paraffin embedding. Prior to staining with H&E (G1120, Solarbio, China), 5‐*μ*m sections were cut and subjected to dewaxing and rehydration. Light microscopy (CX31, Olympus, Japan) was used for the final histological evaluation of the stained sections.

### 2.18. Immunohistochemistry (IHC)

Tumor specimens were processed for protein localization studies by fixation, paraffin embedding, and sectioning (5 *μ*m). Following standard dewaxing, rehydration, and antigen retrieval procedures, nonspecific sites were blocked with 3% BSA (36101ES25, YEASEN, China). Primary antibody incubation targeting ENPP2 (PA5‐12478, Invitrogen, United States), CD206 (DF4149, Affinity, China), and Ki67 (28074‐1‐AP, Proteintech, China) was performed at 4°C overnight. Postincubation washes with PBS were followed by application of species‐matched secondary antibodies. Signals were developed using a DAB chromogen kit (P0203, Beyotime, China), and the results were visualized by conventional light microscopy (CX31, Olympus, Japan).

### 2.19. TUNEL Staining of Tumor Sections

Deparaffinized and rehydrated tumor sections (5 *μ*m) were subjected to antigen retrieval by treating with proteinase K (20 *μ*g/mL, P0096, Beyotime, China) at 37°C for 20 min to expose the antigen epitopes. After antigen retrieval, sections were rinsed two to three times with PBS (pH 7.4) and then permeabilized with 0.1% Triton X‐100 for 10 min on ice. Sections were incubated with 50‐*μ*L Streptavidin‐HRP at 37°C for 60 min, washed with PBS. Apoptotic nuclei were visualized after DAB development (P0203, Beyotime, China) and hematoxylin counterstaining, and images were captured using a light microscope (CX31, Olympus, Japan).

### 2.20. Quantitative Real‐Time PCR (qRT‐PCR)

Total RNA was extracted from samples using Trizol reagent (R0016, Beyotime, China). The concentration and purity of the isolated RNA were subsequently determined with a NanoDrop spectrophotometer. This RNA served as the template for first‐strand cDNA synthesis, which was performed with a reverse transcription kit (D7168S, Beyotime, China) according to the manufacturer′s protocol. Quantitative PCR (qPCR) amplifications were then conducted on a Q225 real‐time PCR system (Kubo, China). Reactions employed Sybr Green master mix (D7268S, Beyotime, China) and followed the recommended thermal cycling profile: an initial denaturation at 95°C for 2 min, followed by 40 cycles of denaturation at 95°C for 15 s and annealing/extension at 60°C for 30 s. Subsequently, a melt curve analysis was performed by gradually increasing the temperature from 60°C to 95°C, with increments of 0.5°C and a 5‐s hold at each step. Relative quantification was performed using the 2^^−*ΔΔ*Ct^ method [24]

Primer sequences used are as follows:

ENPP2: Forward: 5 ^′^‐TCGCTGTGACAACTTGTGTAAG‐3 ^′^, Reverse: 5 ^′^‐CCAATGCGACTCTCCTTTGC‐3 ^′^; CD86: Forward: 5 ^′^‐CTGCTCATCTATACACGGTTACC‐3 ^′^, Reverse: 5 ^′^‐GGAAACGTCGTACAGTTCTGTG‐3 ^′^; IFN‐*γ*: Forward: 5 ^′^‐TCGGTAACTGACTTGAATGTCCA‐3 ^′^, Reverse: 5 ^′^‐TCGCTTCCCTGTTTTAGCTGC‐3 ^′^; CD206: Forward: 5 ^′^‐GGGTTGCTATCACTCTCTATGC‐3 ^′^, Reverse: 5 ^′^‐TTTCTTGTCTGTTGCCGTAGTT‐3 ^′^; IL‐10: Forward: 5 ^′^‐TCAAGGCGCATGTGAACTCC‐3 ^′^, Reverse: 5 ^′^‐GATGTCAAACTCACTCATGGCT‐3 ^′^; *β*‐actin: Forward: 5 ^′^‐CACTCTTCCAGCCTTCCTTC‐3 ^′^, Reverse: 5 ^′^‐GTACAGGTCTTTGCGGATGT‐3 ^′^.

### 2.21. Western Blot Analysis

Cellular proteins were harvested using RIPA lysis buffer (C0045, Targetmol, China), and their concentrations were determined via a bicinchoninic acid (BCA) protein assay kit (20200ES76, YEASEN, China). Equal amounts of protein from each sample were combined with 5× loading buffer (RM00001, Abclonal, China) and denatured at 95°C for 10 min. The denatured samples were then subjected to electrophoresis on a precast polyacrylamide gel (LK302, Epizyme, China). Subsequently, the separated proteins were transferred onto a polyvinylidene difluoride (PVDF) membrane (IPVH00010, Merck Millipore, United States) using a constant current setup. Prior to antibody probing, the membrane was blocked to prevent nonspecific binding. It was then incubated with specific primary antibodies (details provided in Table 1) at 4°C overnight. After extensive washing to remove unbound primary antibodies, the membrane was treated with a horseradish peroxidase (HRP)‐conjugated secondary antibody (A0208, Beyotime, China; diluted 1:5000) for 2 to 4 h at room temperature. Target protein bands were finally detected by applying an enhanced chemiluminescence (ECL) substrate (P0018M, Beyotime, China) to the membrane. The resulting chemiluminescent signals were captured and documented using an SCG‐W2000 imaging system (Servicebio, China).

**Table 1:** Primary antibodies used for western blotting.

Name	Supplier	Catalog no.	Dilution
ENPP2	Invitrogen	PA5‐12478	1:1000
E‐cadherin	Affinity	AF0131	1:1000
N‐cadherin	Affinity	AF5239	1:1000
Vimentin	Affinity	AF7013	1:1000
Bax	Affinity	AF0120	1:1000
Bcl2	Affinity	AF6139	1:1000
Cleaved‐caspase3	Affinity	AF7022	1:1000
Caspase3	Affinity	AF6311	1:1000
CD86	Affinity	DF6332	1:1000
IFN‐*γ*	Affinity	DF6045	1:1000
CD206	Affinity	DF4149	1:1000
IL‐10	Affinity	DF6894	1:1000
Flag	Affinity	T0053	1:1000
GST	Affinity	DF6514	1:1000
*β*‐actin	Abcam	Ab8227	1:5000

### 2.22. Statistical Analysis

A significance threshold of *p* < 0.05 was applied to all analyses. Quantitative data, derived from a minimum of three independent biological replicates (each with three technical replicates), are presented as the mean ± standard deviation. For comparisons involving two experimental groups, Student′s *t*‐test was utilized. Comparisons across multiple groups were conducted using one‐way analysis of variance (ANOVA), followed by Tukey′s test for post hoc analysis.

## 3. Results

### 3.1. ENPP2 Expression Is Increased in HCC Tissues and Correlates With M2 Macrophage Infiltration

**Figure 1:**
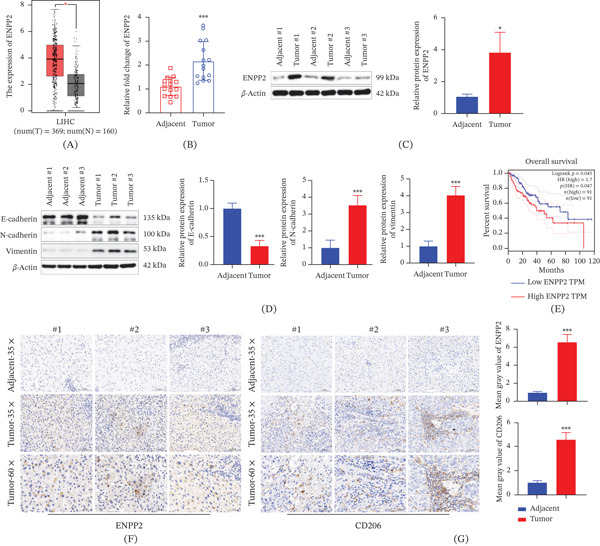
ENPP2 expression level and M2‐type macrophage infiltration in HCC. (A) Analysis of ENPP2 expression levels in HCC using the GEPIA database. (B) The mRNA level of ENPP2 in HCC tissues. (C) The protein level of ENPP2 in HCC tissues. (D) EMT‐related protein expression levels in HCC tissues. (E) Prognostic analysis of ENPP2 using the GEPIA database. (F) Analysis of ENPP2 expression levels in HCC tissues using IHC. (G) Analysis of CD206 expression levels in HCC tissues using IHC. All experiments were performed with three independent biological replicates (each with three technical replicates). Statistical comparisons between two groups were performed using Student′s *t*‐test. ∗*p* < 0.05, ∗∗∗*p* < 0.001, compared to adjacent.

Bioinformatics evaluation via the GEPIA database showed that ENPP2 transcript levels are higher in HCC than in normal liver samples (Figure 1A). Our own clinical samples confirmed this pattern, demonstrating that ENPP2 was markedly overexpressed in tumor tissues at both the mRNA and protein levels relative to paired adjacent noncancerous tissues (Figure 1B,C). Furthermore, profiling of epithelial–mesenchymal transition (EMT) markers in patient samples identified a characteristic shift in HCC, with reduced E‐cadherin and increased N‐cadherin and vimentin expression (Figure 1D). Importantly, survival analysis using GEPIA data revealed that elevated ENPP2 expression correlated with significantly worse overall patient outcomes (Figure 1E). These findings confirm that ENPP2 is overexpressed in HCC. IHC staining of clinical samples demonstrated stronger ENPP2 and CD206 (an M2 macrophage marker) immunoreactivity in tumor areas compared to adjacent tissue (Figure 1F,G). Therefore, the observed pattern implies that the HCC microenvironment is enriched with M2‐polarized TAMs when ENPP2 is highly expressed.

### 3.2. ENPP2 Is a Critical Regulator of Malignant Biological Process and TAM Polarization in HCC

**Figure 2:**
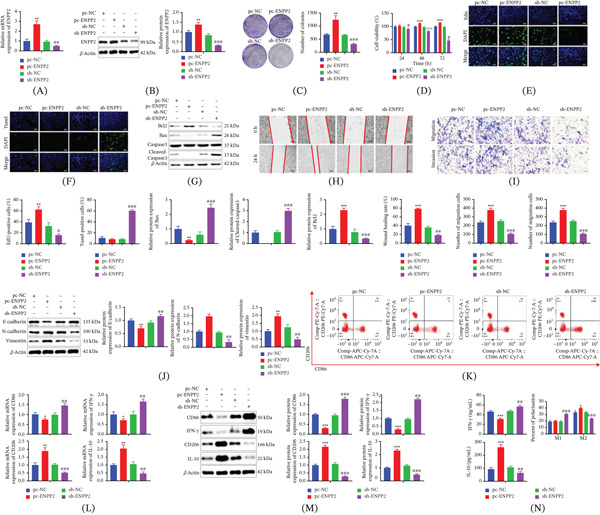
Effect of ENPP2 on the malignant biological process and TAM polarization of HCC. (A) qRT‐PCR assay for transfection efficiency. (B) WB assay for transfection efficiency. (C) Number of HCC cell clone formation. (D) Detection of HCC cell viability using CCK‐8 assay. (E) Analysis of proliferative capacity of HCC cells using EdU fluorescence. (F) Analysis of apoptosis levels in HCC cells using TUNEL fluorescence. (G) The protein levels of apoptosis‐related proteins. Each experiment was replicated three times. (H) Wound repair assay utilized to detect cellular metastatic capacity. (I) Cell migration and invasion detected using the Transwell. (J) The protein levels of EMT‐related proteins. (K) Detection of macrophage polarization ratio by flow cytometry. (L) The mRNA level of macrophage polarization marker. (M) The protein abundance of macrophage polarization marker. (N) Secretion levels of M1‐related cytokine IFN‐*γ* and M2‐related cytokine IL‐10 detected by ELISA. All experiments were performed with three independent biological replicates (each with three technical replicates). Statistical comparisons between two groups were performed using Student′s *t*‐test, and multiple group comparisons were performed using one‐way ANOVA followed by Tukey′s post hoc test. ∗*p* < 0.05, ∗∗*p* < 0.01, ∗∗∗*p* < 0.001, compared to pc‐NC; #*p* < 0.05, ##*p* < 0.01, ###*p* < 0.001, compared to sh‐NC.

To elucidate ENPP2′s functional roles, we conducted gain‐ and loss‐of‐function experiments via transfection with an ENPP2‐overexpressing construct or a targeted shRNA. The efficacy of transfection was confirmed by qRT‐PCR and western blotting. qRT‐PCR results showed that the mRNA level of ENPP2 was significantly upregulated in the pc‐ENPP2 group and significantly downregulated in the sh‐ENPP2 group compared with their respective control groups (Figure 2A). As evidenced by western blotting, ENPP2 protein abundance was significantly higher in the pc‐ENPP2 group than in the pc‐NC control group, whereas sh‐ENPP2 transfection resulted in a substantial knockdown of ENPP2 (Figure 2B). Clonogenic assays revealed that ENPP2 overexpression significantly enhanced the colony‐forming capacity of HCC cells, whereas its knockdown produced the opposite effect (Figure 2C). Functional assays (CCK‐8 and EdU) confirmed that ENPP2 overexpression enhanced proliferative capacity, whereas its knockdown potently suppressed it (Figure 2D,E). Evaluation of apoptosis using the TUNEL assay demonstrated a low frequency of positive cells in the pc‐ENPP2 group relative to controls. The sh‐ENPP2 group conversely exhibited a significant elevation in the number of apoptotic cells (Figure 2F). Corroborating these observations, western blotting of apoptosis regulators detected downregulation of pro‐apoptotic proteins (Bax and cleaved caspase‐3) in pc‐ENPP2 cells and their upregulation following ENPP2 silencing. Bcl‐2, an anti‐apoptotic protein, presented an opposite trend (Figure 2G).

We next investigated the role of ENPP2 in HCC cell metastasis. A wound‐healing assay indicated that ENPP2 overexpression accelerated scratch closure, whereas its inhibition impeded wound repair (Figure 2H). Consistent findings were obtained from Transwell assays: ENPP2 knockdown attenuated the migratory and invasive capacities of HCC cells, whereas its overexpression enhanced these properties (Figure 2I). The molecular profile obtained by immunoblotting further substantiated these functional outcomes. Specifically, heightened ENPP2 expression corresponded to downregulated E‐cadherin and upregulated levels of N‐cadherin and vimentin. Conversely, ENPP2‐silenced cells displayed the opposite expression profile of these EMT‐related proteins (Figure 2J). Together, these results demonstrate that ENPP2 expression critically regulates both proliferative and metastatic capacities in HCC cells.

The polarization status of macrophages is a critical determinant of tumor progression. To determine whether ENPP2, in addition to modulating HCC cell proliferation and metastasis, also influences the polarization of TAMs, we employed a noncontact coculture system as illustrated in Figure S1. Briefly, THP‐1‐derived macrophages were seeded in the lower chamber of six‐well plates, whereas genetically modified HCC cells were plated in the upper Transwell insert at a ratio of 4:1 (HCC cells to macrophages). To determine whether ENPP2, in addition to modulating HCC cell proliferation and metastasis, also influences the polarization of TAMs, we employed a coculture system. A coculture system was established featuring HCC cells (with genetically manipulated ENPP2 levels) and macrophages differentiated from THP‐1 monocytes via PMA treatment. As illustrated in Figure 2K, under control conditions (pc‐NC and sh‐NC), TAMs exhibited a higher proportion of M2‐type relative to M1‐type macrophages. Overexpression of ENPP2 in HCC cells further skewed the polarization balance, reducing the M1 population while significantly increasing the M2 fraction. Conversely, inhibition of ENPP2 reversed this trend, favoring an M1‐dominant phenotype. In line with this functional shift, corresponding analysis of polarization‐specific markers showed that ENPP2 overexpression drove macrophages toward an M2‐skewed phenotype. Molecularly, this change was reflected in a distinct expression pattern: a notable rise in the M2 markers CD206 and IL‐10, coupled with a significant decline in the characteristic M1 markers CD86 and IFN‐*γ*. The opposite expression pattern was observed upon ENPP2 knockdown (Figure 2L,M). ELISA results showed that the secretion level of M1‐related cytokine IFN‐*γ* was significantly decreased, whereas the secretion level of M2‐related cytokine IL‐10 was significantly increased in the pc‐ENPP2 group compared with the pc‐NC group (Figure 2N). In contrast, the sh‐ENPP2 group showed a significant increase in IFN‐*γ* secretion and a significant decrease in IL‐10 secretion compared with the sh‐NC group (Figure 2N). Together, these data indicate that ENPP2 not only drives HCC cell proliferation and metastasis but also concurrently modulates the polarization state of TAMs.

### 3.3. ENPP2 Influences cAMP Expression

**Figure 3:**
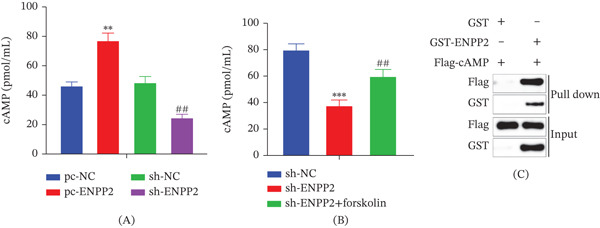
Interaction between ENPP2 and cAMP. (A) The content of cAMP. (B) The concentration of cAMP in different group. (C) GST pull‐down assay to detect the relationship between ENPP2 and cAMP. All experiments were performed with three independent biological replicates (each with three technical replicates). Statistical comparisons between two groups were performed using Student′s *t*‐test, and multiple group comparisons were performed using one‐way ANOVA followed by Tukey′s post hoc test. ∗∗*p* < 0.01, ∗∗∗*p* < 0.001, compared to pc‐NC or sh‐NC; ##*p* < 0.01, compared to sh‐NC or sh‐ENPP2.

Our earlier findings demonstrated that suppressing ENPP2 curbs HCC cell proliferation and metastasis. To elucidate the underlying mechanism, we first measured intracellular cAMP levels. Compared with pc‐NC controls, cAMP concentration was markedly elevated in pc‐ENPP2‐transfected HCC cells, whereas ENPP2 knockdown significantly reduced cAMP content (Figure 3A). The inhibitory effect of sh‐ENPP2 on cAMP was effectively rescued by treatment with forskolin, a known cAMP inducer (Figure 3B). A GST pull‐down assay was performed to explore the potential association between ENPP2 and cAMP‐associated components, using a recombinant GST‐ENPP2 protein and a Flag‐tagged cAMP‐binding probe. The results supported a biochemical association between these two molecules (Figure 3C).

### 3.4. ENPP2 Exerts Its Effects via cAMP Signaling

**Figure 4:**
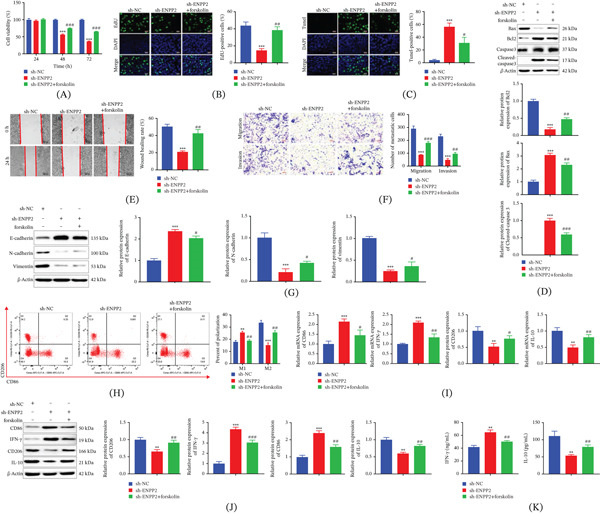
Effect of ENPP2 on proliferation, metastasis, and TAM polarization of HCC via cAMP. (A) The viability of HCC cells. (B) The proliferative capacity of HCC cells. (C) The apoptosis rate of HCC cells. (D) Expression abundance of apoptotic proteins. (E) Cell metastatic ability detected using scratch assay. (F) Cell migration and invasion were detected by utilizing Transwell. (G) The protein levels of E‐cadherin, N‐cadherin, and vimentin. (H) The polarization ratio of TAMs. (I and J) Expression levels of macrophage polarization markers. (K) Secretion levels of M1‐related cytokine IFN‐*γ* and M2‐related cytokine IL‐10 detected by ELISA. All experiments were performed with three independent biological replicates (each with three technical replicates). Statistical comparisons between two groups were performed using Student′s *t*‐test, and multiple group comparisons were performed using one‐way ANOVA followed by Tukey′s post hoc test. ∗∗*p* < 0.01, ∗∗∗*p* < 0.001, compared to sh‐NC; #*p* < 0.05, ##*p* < 0.01, ###*p* < 0.001, compared to sh‐ENPP2.

To investigate the functional relevance of this interaction, sh‐ENPP2‐transfected cells were treated with the cAMP inducer forskolin. The impaired proliferation resulting from ENPP2 knockdown, as measured by CCK‐8 and EdU assays, was largely rescued by forskolin cotreatment (Figure 4A,B). Consistently, the elevated apoptotic rate induced by ENPP2 knockdown was markedly reduced by forskolin (Figure 4C). The alterations in apoptotic regulators observed in sh‐ENPP2 cells, elevated Bax and Cleaved‐caspase3 and reduced Bcl‐2, were negated by the addition of forskolin, as confirmed by western blot analysis (Figure 4D).

The contribution of cAMP to cell motility was subsequently investigated. Functional assays (scratch‐wound and Transwell) demonstrated that the migration and invasion deficits in sh‐ENPP2 cells were partially rescued by forskolin treatment (Figure 4E,F). Administration of forskolin restored the expression of EMT markers, counteracting the molecular profile induced by ENPP2 knockdown, namely, the rise in E‐cadherin and fall in N‐cadherin and vimentin (Figure 4G). Together, these findings establish cAMP signaling as the central mediator through which ENPP2 regulates HCC cell proliferation, apoptosis, and metastatic progression.

Finally, we asked whether cAMP also participates in ENPP2‐driven TAM polarization. Using the same coculture system, flow cytometry analysis revealed that sh‐ENPP2 HCC cells promoted a shift toward M1‐like macrophages, an effect that was counteracted by forskolin (Figure 4H). Concordantly, mRNA and protein levels of M1 markers (CD86 and IFN‐*γ*) were elevated, and M2 markers (CD206 and IL‐10) were reduced in macrophages cocultured with sh‐ENPP2 cells, and these changes were again reversed by forskolin (Figure 4I,J). ELISA showed that forskolin treatment significantly reversed the increase in IFN‐*γ* secretion and decrease in IL‐10 secretion induced by ENPP2 knockdown (Figure 4K). Thus, cAMP signaling not only mediates ENPP2‐dependent HCC cell malignancy but also contributes to its influence on TAM polarization.

### 3.5. In Vivo Tumor Growth Is Promoted by ENPP2 via cAMP Signaling

**Figure 5:**
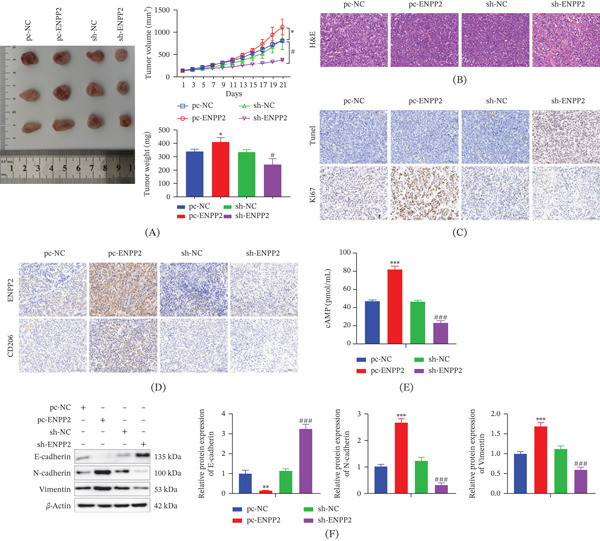
Effects of ENPP2 on xenograft tumors. (A) Anatomical drawings of subcutaneous tumors and analysis of tumor weight and tumor volume. (B) HE staining analysis of subcutaneous tumor tissue lesions. (C) Analysis of apoptosis and proliferative capacity in subcutaneous tumor tissues. (D) IHC analysis of the expression levels of CD206 and ENPP2. (E) The content of cAMP. (F) WB detection of EMT‐related protein expression in subcutaneous tumor tissues. All experiments were performed with three independent biological replicates (each with three technical replicates). Statistical comparisons between two groups were performed using Student′s *t*‐test, and multiple group comparisons were performed using one‐way ANOVA followed by Tukey′s post hoc test. ∗*p* < 0.05, ∗∗*p* < 0.01, ∗∗∗*p* < 0.001, compared to pc‐NC; #*p* < 0.05, ###*p* < 0.001, compared to sh‐NC.

Our in vivo findings indicate that ENPP2 overexpression enhances tumor growth. Xenograft tumors derived from pc‐ENPP2 cells proliferated more rapidly and reached larger dimensions and weights than control (pc‐NC) tumors. Conversely, growth was substantially inhibited in tumors from sh‐ENPP2 cells (Figure 5A). H&E staining revealed tightly packed cells with normal nuclei in pc‐NC, pc‐ENPP2, and sh‐NC tumors, whereas sh‐ENPP2 tumors displayed looser architecture, inflammatory infiltration, and nuclear degeneration (Figure 5B). TUNEL staining indicated abundant apoptosis in sh‐ENPP2 tumors but minimal apoptosis in pc‐ENPP2 tumors (Figure 5C). IHC analysis confirmed higher expression of ENPP2, CD206, and the proliferation marker Ki67 in pc‐ENPP2 tumors compared to controls; sh‐ENPP2 tumors showed opposite trends (Figure 5C,D). cAMP levels were elevated in pc‐ENPP2 tumors and reduced in sh‐ENPP2 tumors (Figure 5E). Western blotting of tumor lysates mirrored the in vitro EMT findings: pc‐ENPP2 tumors had low E‐cadherin and high N‐cadherin/vimentin, a pattern reversed in sh‐ENPP2 tumors (Figure 5F). This in vivo evidence strengthens the relevance of the ENPP2/cAMP pathway in promoting HCC development.

## 4. Discussion

Despite advances in clinical management, HCC remains a deadly malignancy in China, primarily due to late diagnosis, inadequate surveillance biomarkers, suboptimal response to targeted/immunotherapies, and lack of predictive marker [24–30]. This underscores the urgent need for new molecular targets and biomarkers to improve HCC management.

Accumulating evidence underscores a critical oncogenic function for the secreted glycoprotein ENPP2. In chronic lymphocytic leukemia (CLL), for example, ENPP2 is frequently dysregulated. Its abrogation via gene silencing or the inhibitor PF‐8380 not only restrained tumor proliferation but also potentiated response to ibrutinib, suggesting a role in metabolic reprogramming and supporting ENPP2 as a viable target [31]. Our work demonstrates that ENPP2 is significantly upregulated in HCC tissues, correlating with poorer patient prognosis and M2 TAM infiltration—an association that has not been previously linked to ENPP2 in HCC. Functionally, suppressing ENPP2 in HCC cells impeded their proliferative and metastatic capacities, a finding consistent with prior reports [32, 33], but our study identifies that ENPP2 modulation in HCC cells directly drives TAM polarization via cAMP signaling, a mechanistic link that distinguishes our work from existing literature on TAM modulation in HCC. Specifically, ENPP2‐overexpressing HCC cells drove cocultured macrophages toward a protumor M2 phenotype, whereas ENPP2 inhibition promoted an antitumor M1 state, and cAMP was confirmed as the critical intracellular mediator of this crosstalk. This positions ENPP2 not only as a direct promoter of HCC cell aggression but also as a key regulator of tumor‐immune crosstalk via the ENPP2/cAMP/TAM axis, opening avenues for combining ENPP2‐targeted approaches with immunotherapy in HCC management.

The polarization state of TAMs is a critical immunological determinant influencing neoplastic growth within the TME [34]. Strategies to reprogram TAMs are actively explored. For example, *Chen* and colleagues demonstrated that inhibiting gasdermin E (GSDME) in nonmalignant cells reduced M2 TAM prevalence in the HCC TME and boosted CD8^+^ T cell cytotoxicity, suggesting a promising combinatory immunotherapeutic strategy [35]. The long noncoding RNA MEG3 has been shown to exert immunomodulatory effects in HCC. *Lin* et al. reported that it promotes M1 macrophage polarization via inhibition of colony‐stimulating factor 1 (CSF‐1), thereby altering the tumor immune microenvironment [36]. Additionally, the compound hypericin was shown to inhibit M2 polarization via the phosphoinositide 3‐kinase (PI3K)/AKT pathway, subsequently restraining HCC progression [37]. While these studies collectively affirm the impact of macrophage polarization on HCC, they primarily involve direct manipulation of macrophages. Our study adopts a distinct approach by modulating a tumor cell‐intrinsic factor ENPP2 within an in vitro coculture system that mimics TME interactions. We found that altering ENPP2 expression in HCC cells indirectly but effectively shifted the macrophage polarization balance. Mechanistically, we identified that ENPP2 functions, at least in part, by interacting with and modulating intracellular cAMP levels, as evidenced by GST pull‐down assays and forskolin rescue experiments.

Mechanistically, we identified that ENPP2 modulates intracellular cAMP levels, a signaling pathway implicated in HCC pathogenesis [38–40]. Notably, previous studies have not established a link between ENPP2 and cAMP signaling in HCC, nor have they elucidated how tumor cell‐intrinsic ENPP2 regulates TAM polarization via cAMP. Unlike previous studies that focused on downstream cAMP effects or direct cAMP modulation [38–40], our data novelly demonstrate that ENPP2 acts upstream to leverage cAMP signaling as a core mediator, concurrently promoting HCC cell malignancy and protumorigenic TAM polarization. We used a GST pull‐down assay to explore the potential association between ENPP2 and cAMP‐associated components, which provided preliminary evidence of a biochemical association, but we acknowledge several limitations of this approach. First, ENPP2 is primarily an extracellular enzyme, whereas cAMP is a small intracellular secondary messenger (329 Da), making a direct physical interaction between the two molecules unusual. Thus, the observed association likely reflects an indirect interaction—such as ENPP2 binding to a cAMP‐binding protein or a downstream mediator of cAMP signaling—rather than direct binding to cAMP itself. Second, the Flag‐tagged cAMP‐binding probe used in the assay may have altered native conformation and binding properties due to the size of the Flag tag (~1 kDa, approximately three times larger than cAMP), which could introduce bias into the results. Despite these limitations, our forskolin rescue experiments provide strong functional evidence that cAMP signaling is required for ENPP2‐mediated regulation of HCC cell malignancy and TAM polarization, complementing the GST pull‐down data and strengthening the logical connection between ENPP2 and cAMP. Importantly, forskolin only partially rescues the effects of ENPP2 knockdown, which indicates that cAMP signaling is a critical but not the sole mediator of ENPP2′s functions. In addition, since forskolin is a cell‐permeable cAMP activator, we cannot fully exclude potential direct effects on macrophage polarization in the coculture system. However, given that ENPP2 expression was genetically modulated only in HCC cells and that forskolin administration restored cAMP levels primarily in ENPP2‐deficient HCC cells, the observed reversal of TAM polarization strongly supports the functional importance of cAMP signaling in HCC cells as a paracrine mediator of ENPP2‐driven macrophage skewing. Our data therefore indicate that cAMP signaling in HCC cells is necessary for ENPP2‐mediated TAM polarization, even if direct effects of forskolin on macrophages cannot be completely ruled out. This observation suggests the existence of additional, cAMP‐independent pathways that contribute to ENPP2‐driven HCC progression and TAM polarization, an important limitation that we explicitly acknowledge.

Plausible cAMP‐independent mechanisms mediating ENPP2′s functions are consistent with its known biological role as a secreted enzyme that generates LPA. Beyond its effects on cAMP signaling, LPA can activate multiple alternative signaling cascades that are well‐documented to promote HCC progression and macrophage polarization, including the PI3K/AKT, MAPK (ERK1/2, JNK, p38), and Rho GTPase pathways. For instance, LPA‐induced PI3K/AKT activation has been shown to enhance HCC cell proliferation and migration, whereas MAPK signaling can regulate macrophage polarization toward an M2 phenotype. Additionally, ENPP2 may exert direct effects independent of its enzymatic activity and cAMP signaling, such as through interactions with cell surface receptors (e.g., integrins) or extracellular matrix components, which could further modulate HCC cell behavior and TAM polarization. These cAMP‐independent pathways may act in parallel with the ENPP2/cAMP axis to mediate ENPP2′s oncogenic effects, explaining the partial rescue observed with forskolin. Further studies are warranted to validate these potential alternative mechanisms and clarify their relative contributions to ENPP2‐driven HCC progression.

Beyond mechanistic inference, these findings position ENPP2‐centered molecular readouts as a plausible component of biomarker pipelines in HCC, particularly when integrated with transcriptomic, proteomic, and immune‐contexture features derived from public cohorts. For translational use, priority should be given to pre‐analytical control, assay harmonization (e.g., tissue vs. circulating measurements), and transparent reporting of analytical performance (precision, stability, and interlaboratory reproducibility). Given the clinical heterogeneity of HCC, follow‐up validation should be designed across independent, etiologically stratified cohorts and evaluated for robustness in clinically relevant subgroups. In this framework, ENPP2 readouts can be treated as a candidate node within a broader variant‐to‐biomarker path that will ultimately require linking molecular signals to clinically interpretable endpoints.

Several limitations should be acknowledged in the present study. First, the clinical validation cohort consisted of only 10 pairs of HCC tissues. Although consistent trends were observed in this set and were further supported by public database analyses, this sample size is insufficient to establish robust statistical correlations, particularly between ENPP2 expression and M2 macrophage infiltration. The limited cohort also restricts the generalizability of our findings to the broader, etiologically diverse HCC population. Future studies with larger, well‐annotated independent cohorts are warranted to confirm the clinical significance of the ENPP2–cAMP–TAM axis. Second, in vitro experiments were performed using a single HCC cell line (HepG2), which may not fully capture the biological heterogeneity of human HCC. Future studies incorporating additional cell line models (e.g., Huh7 and MHCC97H) will be essential to confirm the generalizability of our observations. Third, while the GST pull‐down assay confirmed a biochemical association between ENPP2 and cAMP‐associated components, the precise molecular mechanism by which ENPP2 regulates intracellular cAMP levels warrants further investigation. One plausible mechanism involves ENPP2‐generated LPA, which may activate specific G‐protein‐coupled receptors (GPCRs) known to modulate adenylate cyclase activity, thereby influencing cAMP production. Alternatively, ENPP2 may indirectly affect cAMP levels through downstream signaling intermediates or lipid metabolism crosstalk. Fourth, the xenograft model employed in this study relies on subcutaneous injection, which does not recapitulate the native liver microenvironment or the immune cell trafficking dynamics characteristic of orthotopic or spontaneous HCC models. This limitation may affect the translational relevance of our in vivo findings, as the subcutaneous microenvironment differs substantially from the liver′s unique anatomical and immunological context; future studies using immunocompetent mouse models with lineage tracing or macrophage depletion strategies are required to establish causality and therapeutic relevance. Fifth, the partial rescue effect of forskolin suggests the involvement of cAMP‐independent pathways in ENPP2′s functions, which require further exploration to fully delineate the comprehensive mechanism of ENPP2 in HCC. Sixth, as highlighted by the reviewer, we have not yet investigated the specific mutations in the ENPP2 protein that lead to its abnormal expression in HCC. ENPP2 mutations have been reported in various malignancies, including breast cancer and lung cancer, where specific missense mutations enhance its enzymatic activity and oncogenic potential. In HCC, the genetic landscape of ENPP2 mutations remains poorly characterized; our preliminary analysis of public HCC sequencing datasets (TCGA‐LIHC) identified rare missense mutations in ENPP2, but their functional impact on ENPP2 expression, enzymatic activity, and ability to regulate macrophage polarization has not been explored. This represents a critical gap, as specific ENPP2 mutations may drive its abnormal upregulation and protumorigenic functions in subsets of HCC patients, potentially serving as predictive biomarkers for ENPP2‐targeted therapies. Seventh, while we explored potential intercellular communication mechanisms (soluble factors, exosomes, and metabolic reprogramming), the relative contribution of each mechanism to ENPP2‐mediated macrophage polarization remains unclear. Future studies using specific inhibitors (e.g., LPA receptor antagonists and exosome secretion inhibitors) will be needed to dissect the hierarchical role of these pathways. Addressing these limitations in future work will be essential to translate these findings toward clinical application.

## 5. Conclusions

In conclusion, ENPP2 is upregulated in HCC and promotes tumor malignancy and M2 macrophage polarization via cAMP signaling. These findings suggest that ENPP2, as a secreted enzyme, holds potential as a noninvasive biomarker for risk stratification and treatment monitoring. The ENPP2–cAMP–TAM axis also represents a candidate therapeutic target that may complement existing immunotherapies by reprogramming the immunosuppressive TME.

## Author Contributions

**S.C. and J.L.:** conceptualization, writing – original draft, data curation. **J.L. and P.O.:** investigation, visualization. **J.T.:** resources. **J.L.:** funding acquisition. **F.L.:** supervision, project administration, writing – review and editing.

## Funding

This study was supported by the Natural Science Foundation of Hunan Province, 10.13039/501100004735, No. 2025JJ81095.

## Disclosure

All authors reviewed and approved the final manuscript.

## Ethics Statement

All procedures involving human participants conformed to the ethical standards of the *Declaration of Helsinki* and were approved by the Ethics Committee of the University of South China (Approval No: 2024‐KY‐170). Informed consent was obtained from all participants. The animal study protocol was approved by the Animal Ethics Committee of the University of South China (Approval No.: 2025‐KY‐304).

## Consent

Informed consent for publication was obtained from all subjects involved in the study.

## Conflicts of Interest

The authors declare no conflicts of interest.

## Supporting information


**Supporting Information** Additional supporting information can be found online in the Supporting Information section. Figure S1: Schematic representation of the noncontact Transwell coculture system. THP‐1‐derived macrophages were seeded in the lower chamber of six‐well plates. HCC cells with stable ENPP2 overexpression or knockdown were plated in the upper Transwell insert (0.4‐*μ*m pore size) at a ratio of 4:1 (HCC cells to macrophages). After 48 h of coculture, macrophages in the lower compartment were harvested for flow cytometry, qRT‐PCR, western blot, and ELISA to assess M1/M2 polarization status.

## Data Availability

The datasets generated and analyzed during the current study are available from the first author, S.C., upon reasonable request.
