# Supplementary figures and images for: ENPP2 Dysregulation Defines a Candidate Biomarker Axis Coupling Tumor‐Intrinsic cAMP Signaling to Macrophage Polarization in Hepatocellular Carcinoma
Source: Hum Mutat. 2026 May 23;2026:9266306. doi: 10.1155/humu/9266306 (PMC13197829; doi:10.1155/humu/9266306)

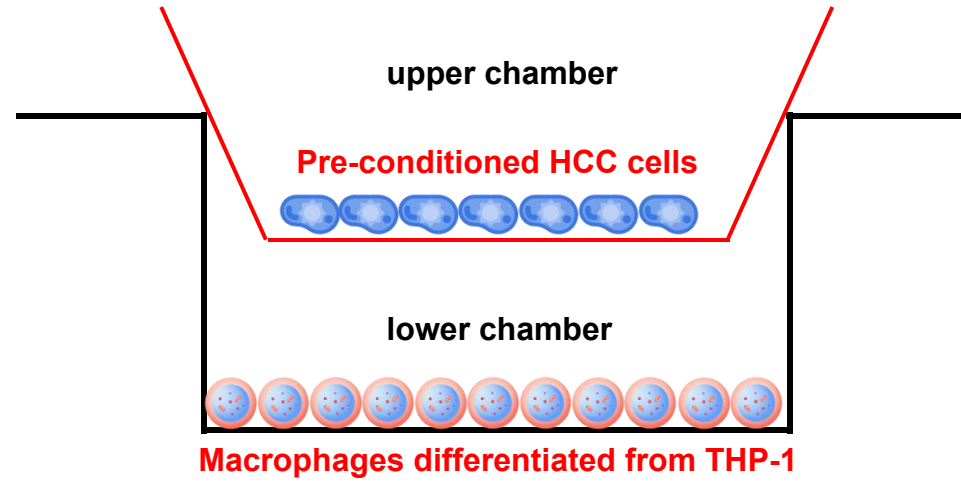

Supplement: Supplementary file 1 — Supporting Information Additional supporting information can be found online in the Supporting Information section. Figure S1: Schematic representation of the noncontact Transwell coculture system. THP‐1‐derived macrophages were seeded in the lower chamber of six‐well plates. HCC cells with stable ENPP2 overexpression or knockdown were plated in the upper Transwell insert (0.4‐μm pore size) at a ratio of 4:1 (HCC cells to macrophages). After 48 h of coculture, macrophages in the lower compartment were harvested for flow cytometry, qRT‐PCR, western blot, and ELISA to assess M1/M2 polarization status. [file HUMU-2026-9266306-s001.pdf]
